# Supplementary material for: The Musashi proteins direct post-transcriptional control of protein expression and alternate exon splicing in vertebrate photoreceptors
Source: Commun Biol. 2022 Sep 24;5:1011. doi: 10.1038/s42003-022-03990-w (PMC9509328; doi:10.1038/s42003-022-03990-w)
Supplement: Supplementary file 2 — Supplementary Information (new) [file 42003_2022_3990_MOESM2_ESM.pdf]

Supplementary Information

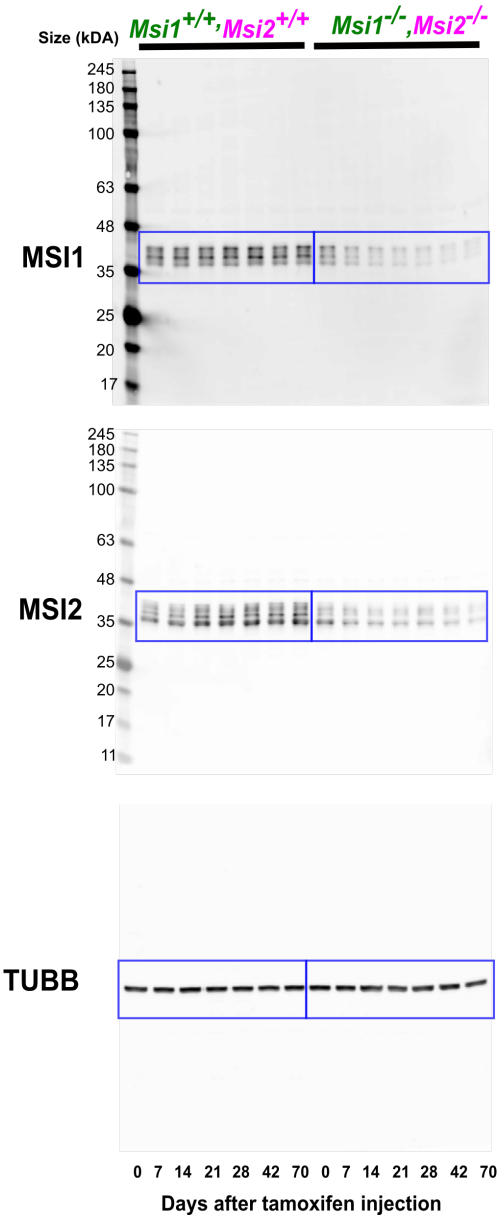

Supplementary Figure 1. Full size western blot images for the data presented on Figure 1b. Labels on the left indicate the protein being probed. Animal genotypes are indicated on the top and the days after tamoxifen injection are indicated at the bottom. Day 0 after tamoxifen injection corresponds to postnatal day 30. Size markers indicate the molecular weight in kDA. Blue boxes show the parts of the blots shown in Figure 1b.

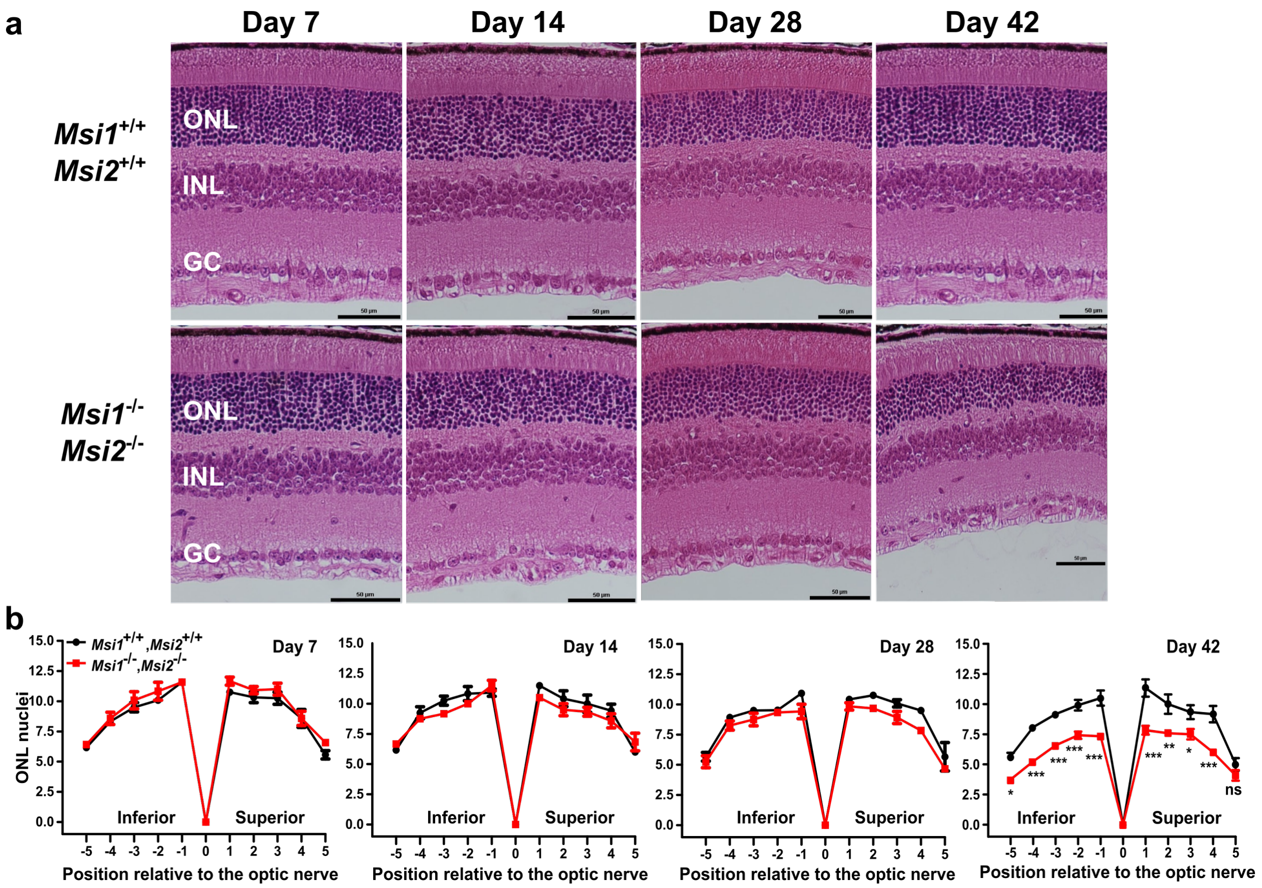

Supplementary Figure 2. Outer nuclear layer thickness of the retina after double knockout of *Msi1* and *Msi2* in photoreceptor cells. (a) Representative H&E-stained eye sections from the double *Msi1*/*Msi2* knockout and age matched controls at 7, 14, 28 and 42 days after inducing the knockout. ONL: outer nuclear layer (Photoreceptor nuclei), INL: inner nuclear layer, GC: ganglion cells. 40X objectives and scale bar represents 50 μm. **(b)** Spider plots displaying the thickness of the ONL as the number of nuclei measured at ten points stepped by 400μm from the optical nerve at different time points post-tamoxifen injection. Data are shown as mean ± SEM. Pairwise t-test with Bonferroni correction for multiple comparisons was used to determine the effect of the genotype on the outer nuclear layer thickness at each time point. Significance levels of the pairwise comparisons is indicated as: * p-value < 0.05, ** p-value < 0.01, *** p-value < 0.001. Supplementary Data 15 contains the source data underlying the graphs.

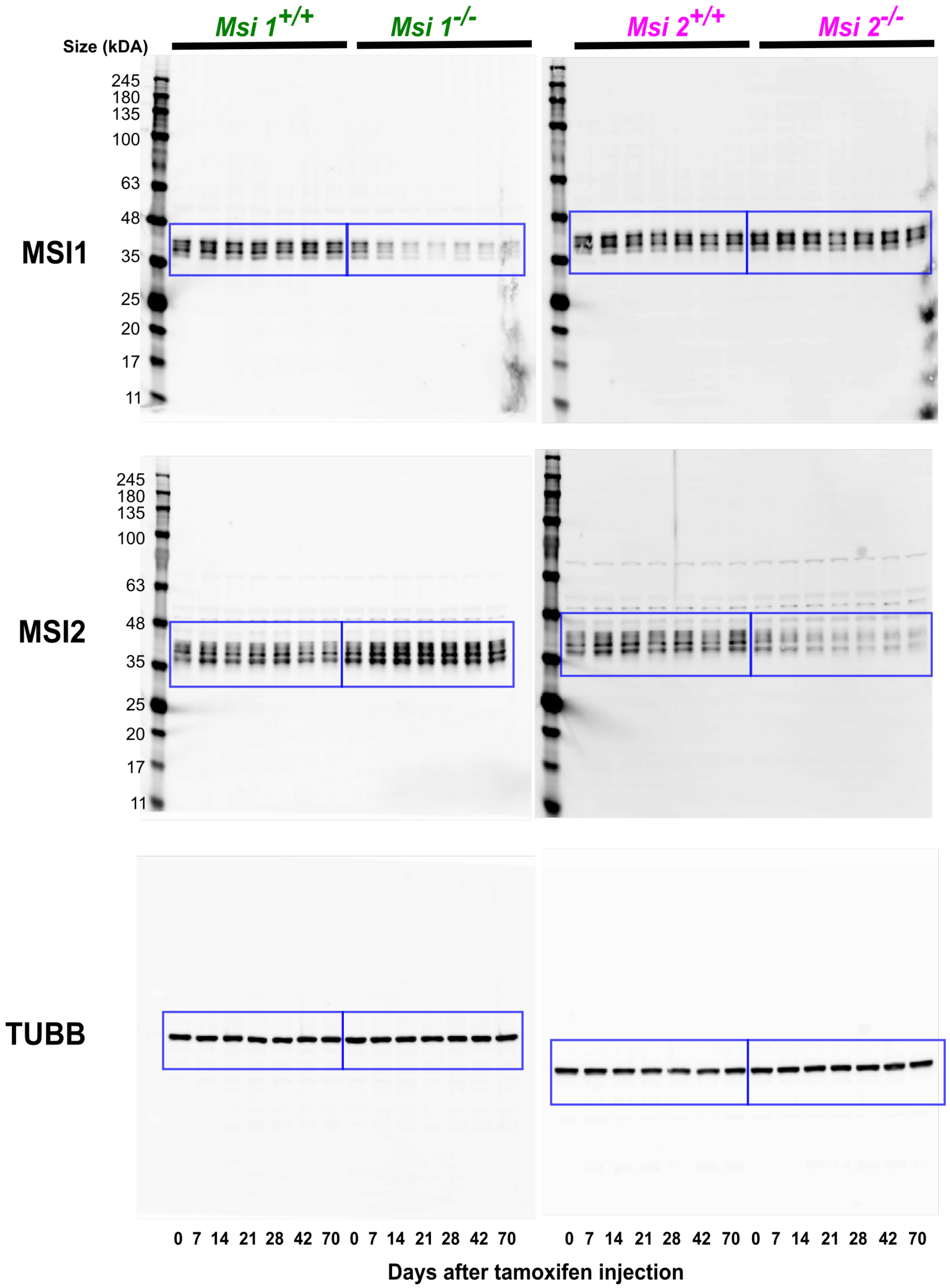

Supplementary Figure 3. Full size western blot images for the data presented on Figure 3b. Labels on the left indicate the protein being probed. Animal genotypes are indicated on the top and the days after tamoxifen injection are indicated at the bottom. Day 0 after tamoxifen injection corresponds to postnatal day 30. Size markers indicate the molecular weight in kDA. Blue boxes

show the parts of the blots shown in Figure 3b.

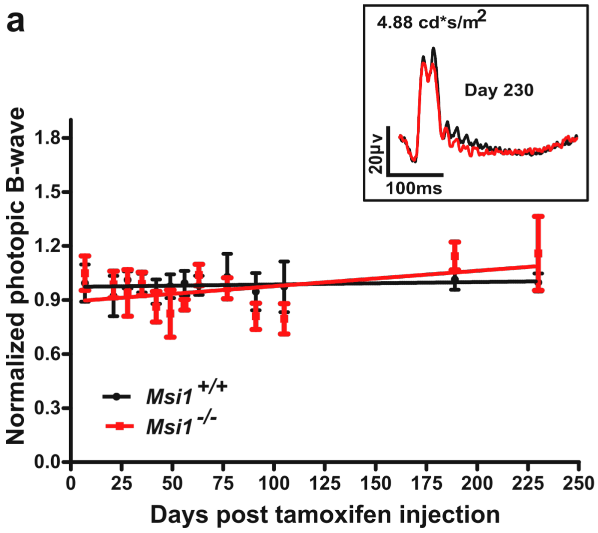

Supplementary Figure 4. Normal photopic response to light in the single *Msi1* or *Msi2* knockouts. Photopic mean B-wave response of knockout (red) *Msi1*$^{-/-}$ **(a)** and *Msi1*$^{-/-}$ **(b)** mice (red line) and age matched controls (black) between day 16 and day 230 post tamoxifen injection. Photopic waveforms were obtained after light adaptation using 4.88 cd-s/m^2 flashes. The insets show representative photopic (light-adapted) electroretinograms recorded 230 days post-injection using 4.88 cd-s/m^2 flashes. The data points from the photopic responses of the single depletion of *Msi1* or *Msi2* are represented as mean ± SEM of 8 eyes, from 4 animals. Supplementary Data 15 contains the source data underlying the graphs.

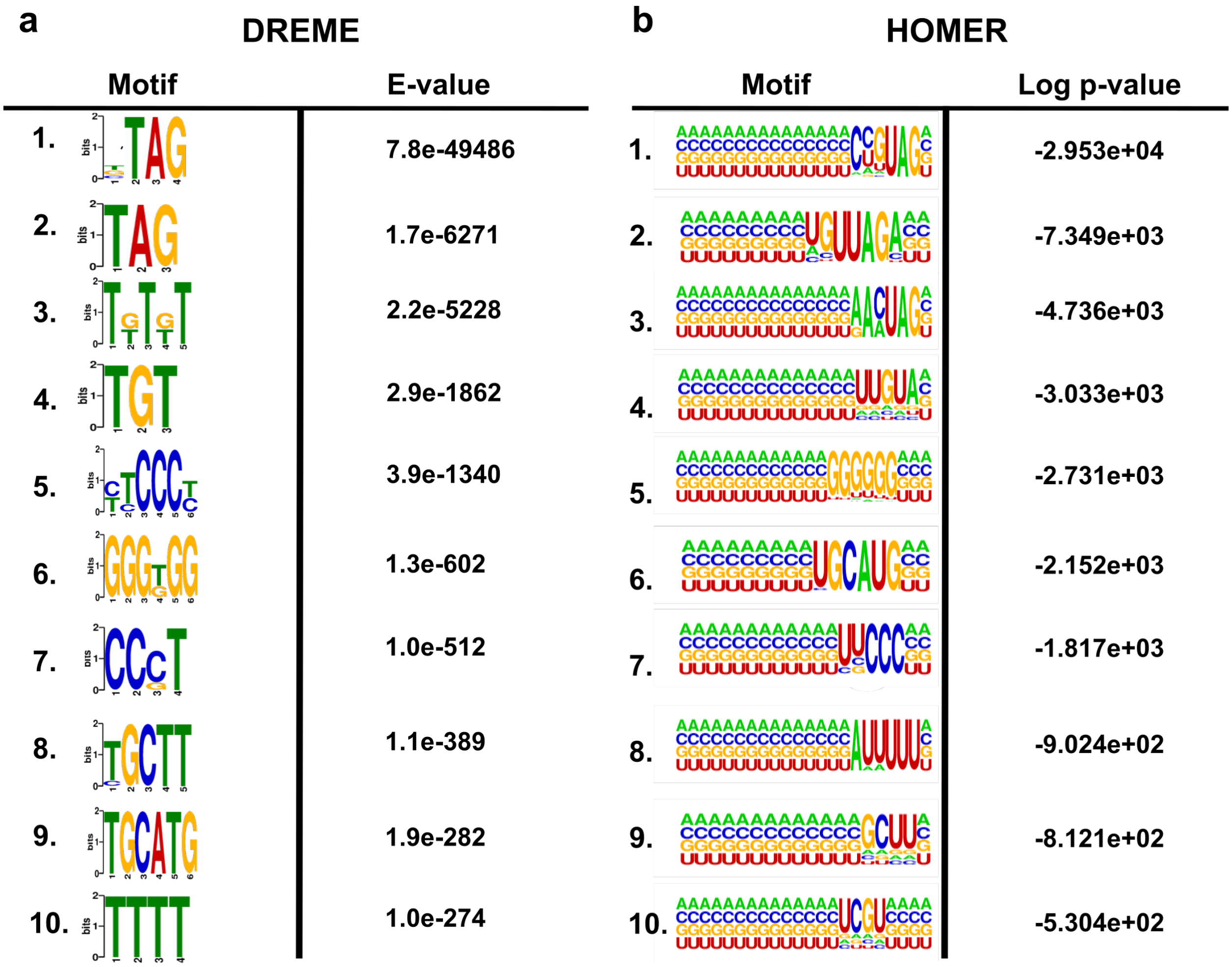

Supplementary Figure 5. Sequence motifs enriched near eCLIP-Seq derived MSI1 crosslinks sites. Logos of the top ten significantly enriched motifs identified by DREME **(a)** or HOMER **(b)** in the vicinity of MSI1 crosslink sites.

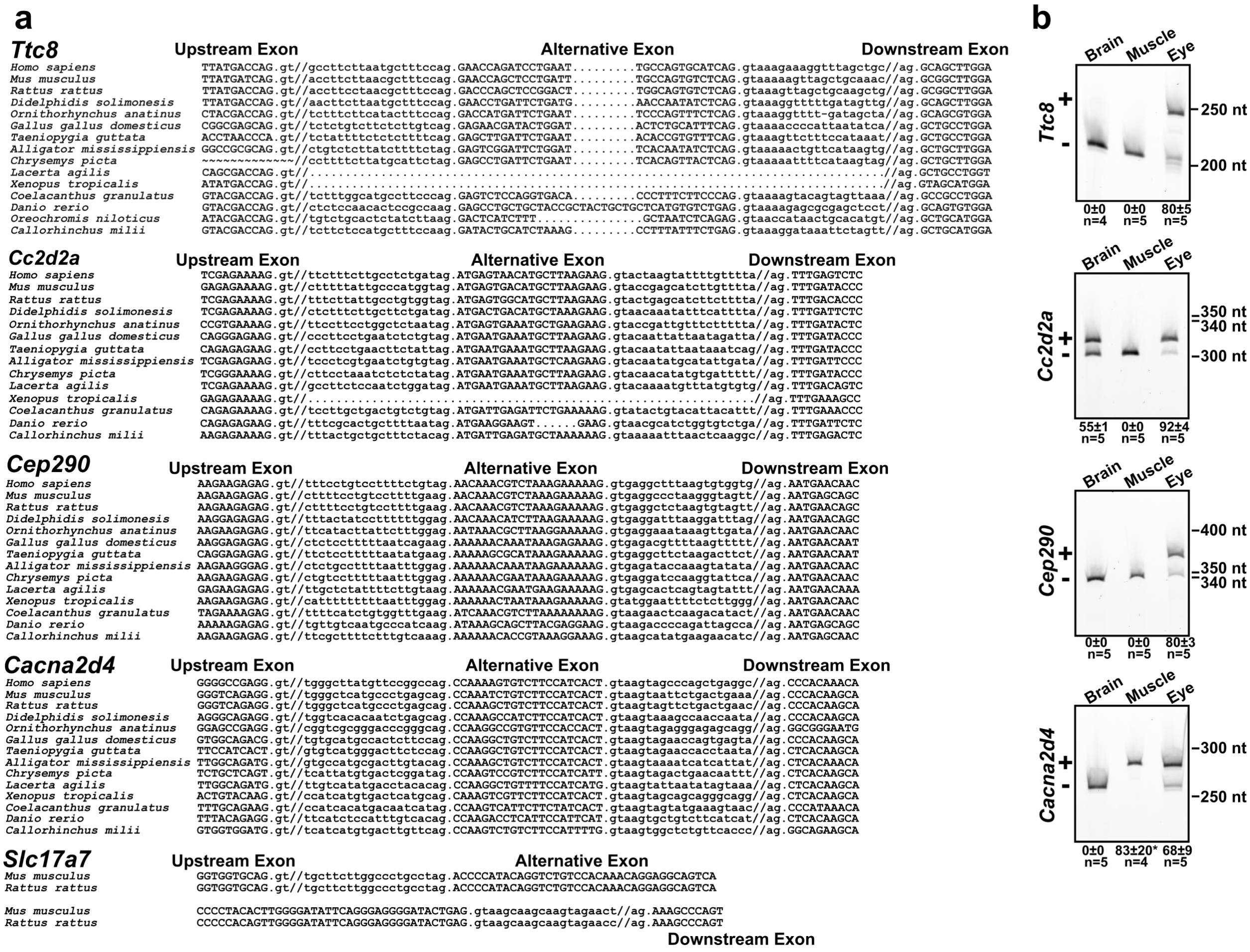

Supplementary Figure 6: Conservation of the photoreceptor-specific exons of *Ttc8, Cc2d2a, Cep290, Cacna2d4, and Slc17a7* across vertebrates. (a) Alignments showing the alternative exons and parts of the flanking constitutive exons. Introns are in lower case and exons are in upper case. Forward slashes indicate where intronic sequences were removed from the alignment for ease of presentation. Homologous exons for the photoreceptor specific exons in *Ttc8, Cc2d2a, Cep290,* and *Cacna2d4* can be traced down to Chondrichthyes. The exons in *Ttc8,* and *Cc2d2a* can vary in length while preserving the reading frame or be completely absent from certain species. The upstream exon is not available for *Chrysemys picta* due to gaps in the genome sequence. The exon in *Slc17a7* is present only in rodents. **(b)** Analysis of the inclusion rate of the zebrafish homologues of the photoreceptor-specific exons in the *Ttc8*, *Cc2d2a*, *Cep290*, and *Cacna2d4* in

brain, muscle, and eye samples. Numbers under the figure indicate the percent inclusion of the exon ± SEM. All four exons have high inclusion rates in the eye. Unlike their mouse homologues the zebrafish exons in the *Cc2d2a* and *Cacna2d4* genes are also included at high rate in the brain and muscle, respectively. Size markers indicate the fragment size in nucleotides. *The exon in *Cacna2d4* was typically included at 100% (3 out of 4 tested samples). Supplementary Data 15 contains the source data underlying the graphs.

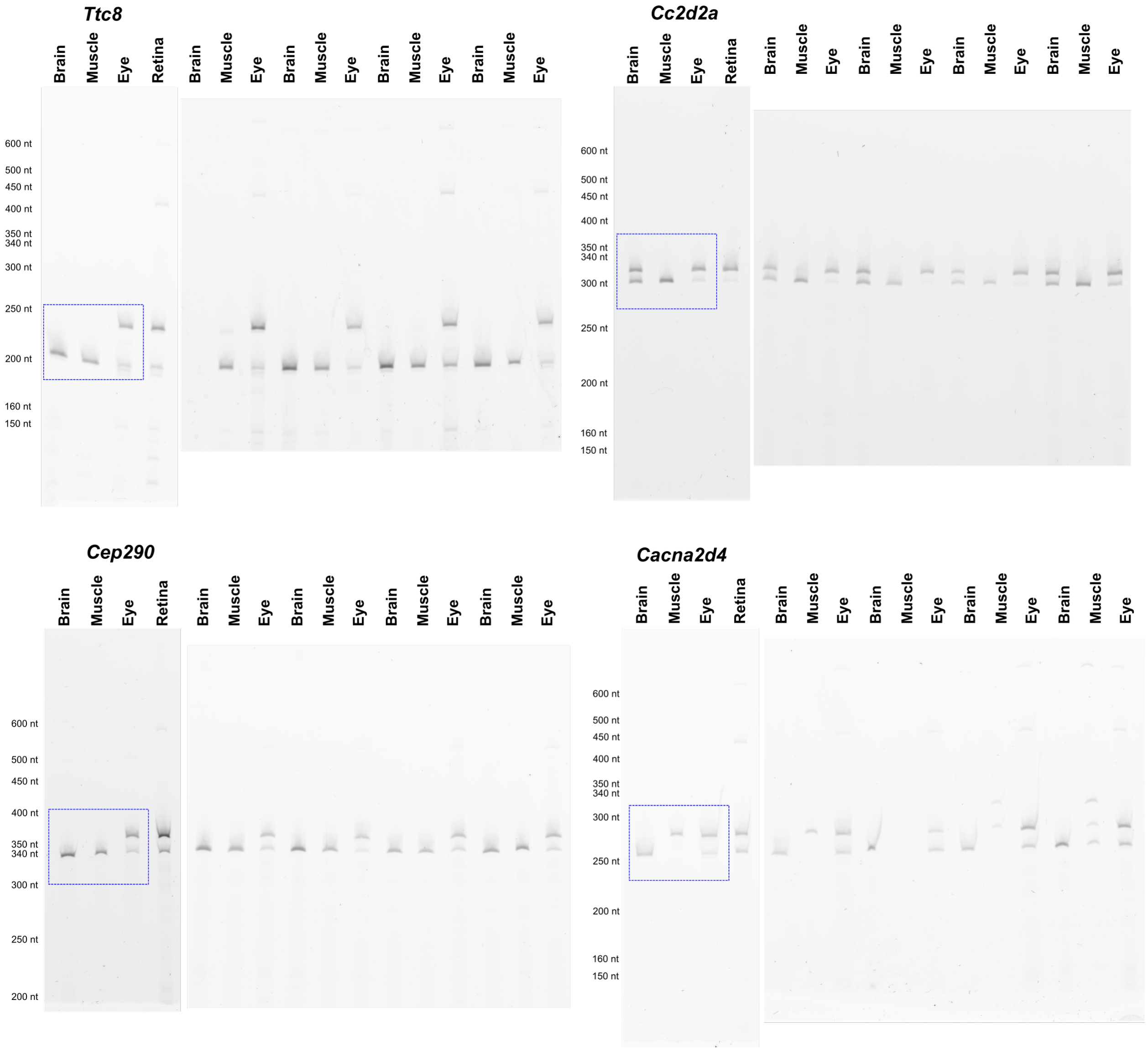

Supplementary Figure 7. RT-PCR gel images underlying the data presented on

Supplementary Figure 6b. Labels on top show the gene in which alternative splicing is being analyzed and the tissue from which the RNA was extracted. Blue boxes represent the parts of the gels shown on Supplementary figure 6b. Additional gels show replicates used to quantify exon inclusion levels.

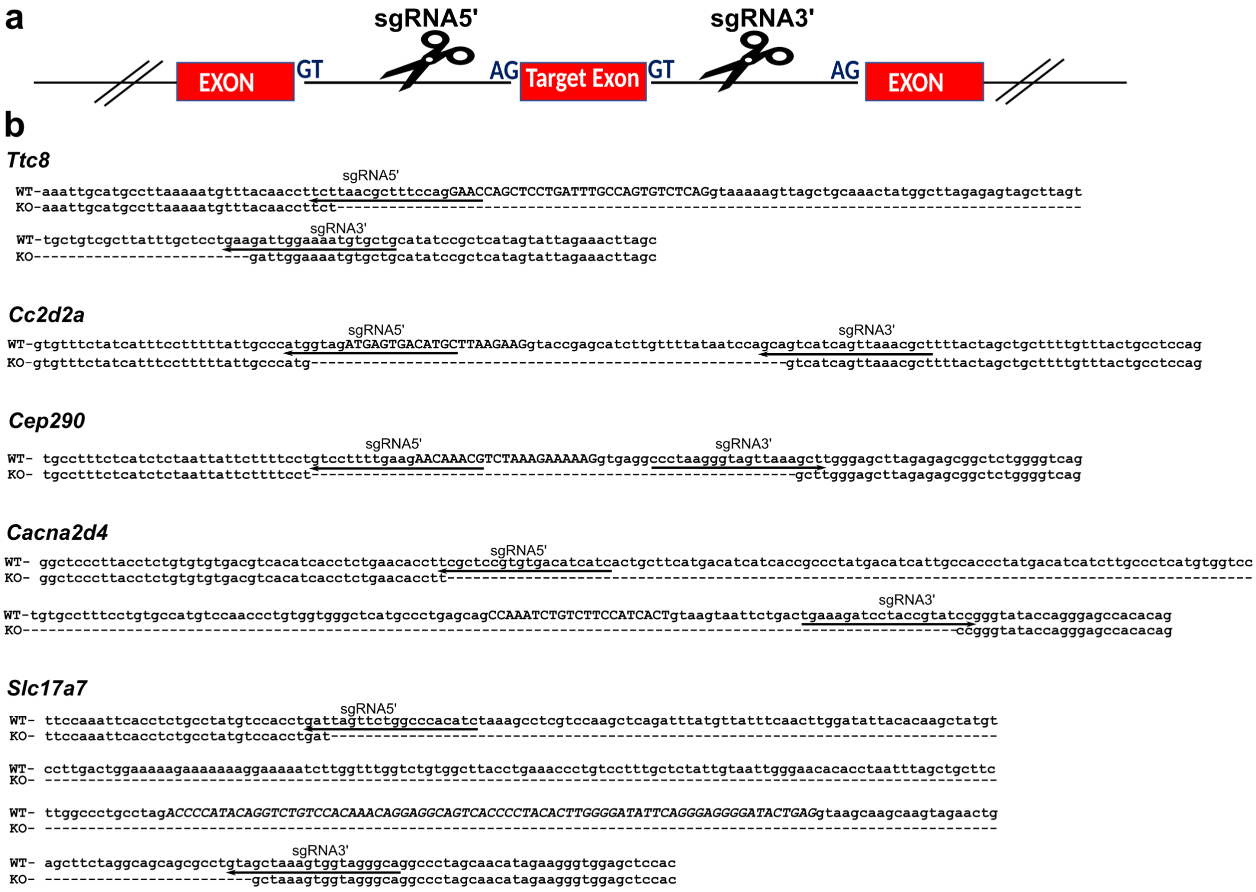

Supplementary Figure 8. Exon deletion alleles generated by CRISPR/Cas9 mutagenesis. (a) Schematic of the CRISPR/Cas9 targeting strategy. Two guide RNAs are used to direct cuts on both sides of the exon leading to its deletion. **(b)** Sequences of the knockout alleles (KO) aligned to the wild type genomic sequence (WT). The exons are shown in uppercase and introns are in lowercase. Arrows indicate the position and orientation (5' to 3') of the guide RNAs.

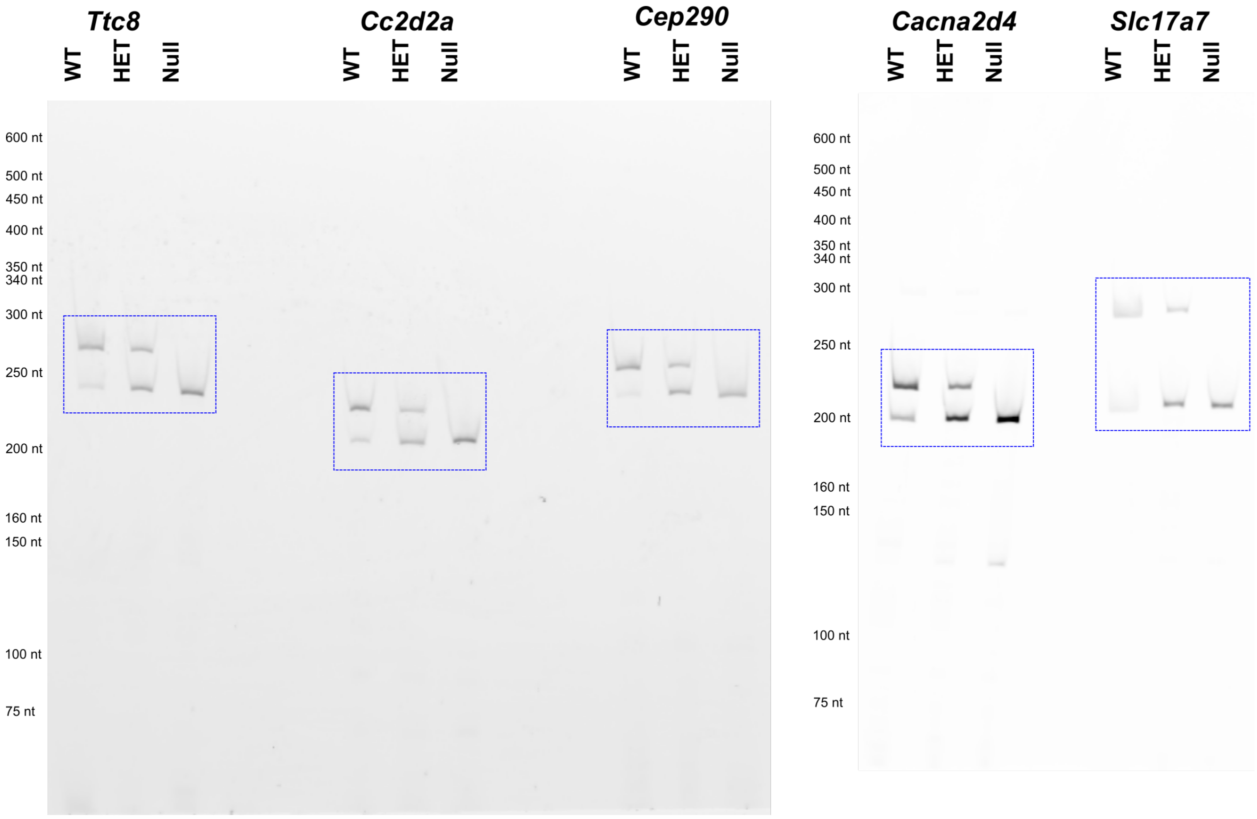

Supplementary Figure 9. RT-PCR gel images underlying the data presented on Figure 6a. Labels on top show the gene in which alternative splicing is being analyzed and the genotype of the animal from which the retinal sample was collected. Blue boxes represent the parts of the gels shown on Supplementary Figure 6b.

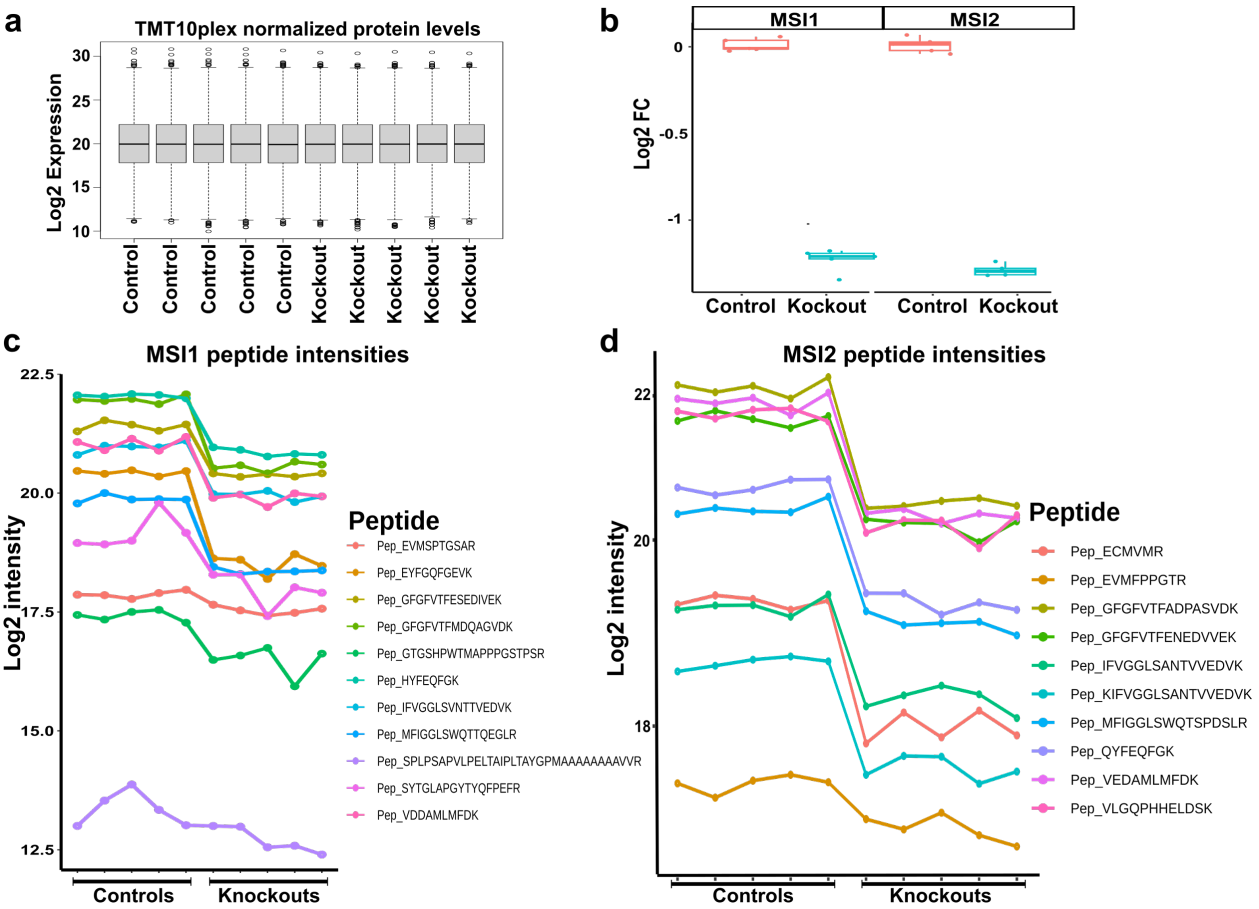

Supplementary Figure 10. Decrease of MSI1 and MSI2 protein levels in the retina after induced double knockout of *Msi1* and *Msi2* in mature photoreceptor cells. (a) Box plot showing the distribution of normalized signal intensities across samples analyzed by isobaric labeling and tandem MS (MS3). **(b)** Box plots showing the log2 of the fold difference of MSI1 and MSI2 protein levels in the retina of control and knockout mice relative to the median of the controls. Hinges of the boxplots on panels a and b represent the range between the first and third quartile. Median is indicated by a line between the hinges. Whiskers extend from the hinges to 1.5 times the inter-quartile range (IQR). On panel a outliers are presented as open circles. On panel b dots represent individual data points. Changes in the levels of individual tryptic peptides identified for MSI1 **(c)** and MSI2 **(d)** in control and knockout retina. Supplementary Data 17 and 18 contain the peptide and protein level source data underlying the graphs.

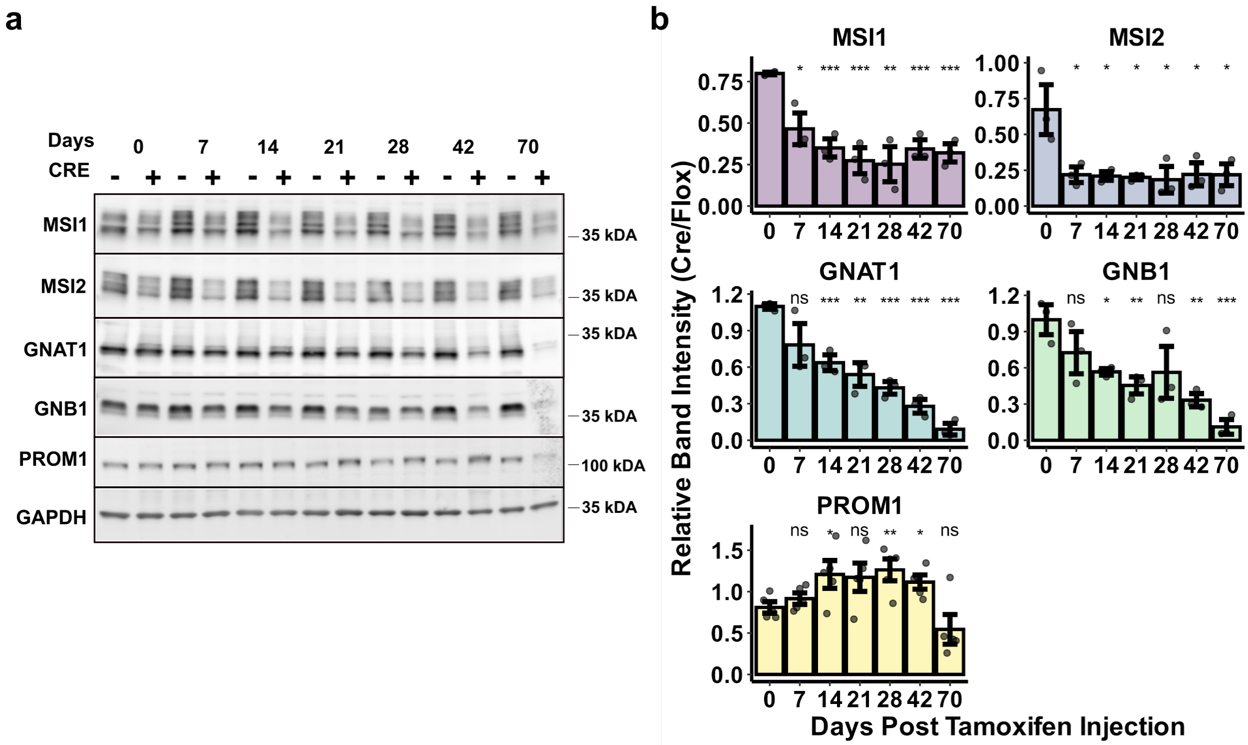

Supplementary Figure 11. **Validation of MS3 data by western blot. (a)** Representative immunoblot showing levels of selected proteins after combined deletion of *Msi1* and *Msi2* in mature photoreceptors. Size markers indicate the molecular weight in kDA. **(b)** Quantification of western blot data for MSI1, MSI2, GNAT1, GNB1 and PROM1. TUBB1 and GAPDH were used as controls to normalize for loading. Error bars represent standard error of the mean (SEM, n=3). The statistical significance of the pairwise comparisons of the protein levels at different time points to the baseline level at the day of the tamoxifen injection is indicated as: * p-value < 0.05, ** p-value < 0.01, *** p-value < 0.001. Supplementary Data 15 contains the source data underlying the graphs.

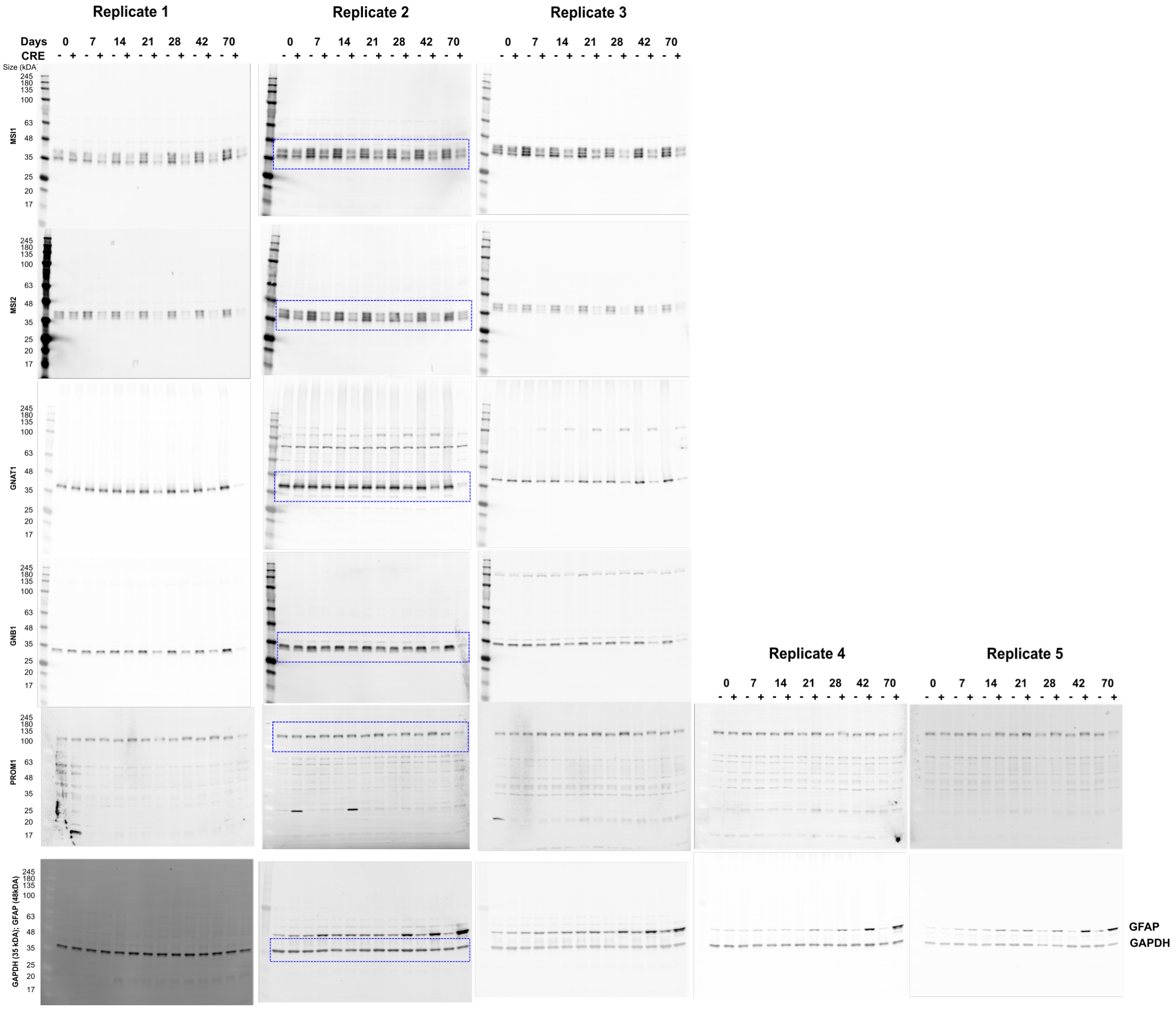

Supplementary Figure 12. Full size western blot images for the data presented on Supplementary Figure 11. Gels are grouped in replicates. Each replicate represents one independent set of samples. Labels on the left indicate the protein being probed. Size markers indicate the molecular weight in kDA. Blue boxes show the parts of the blots shown on Supplementary Figure 11.

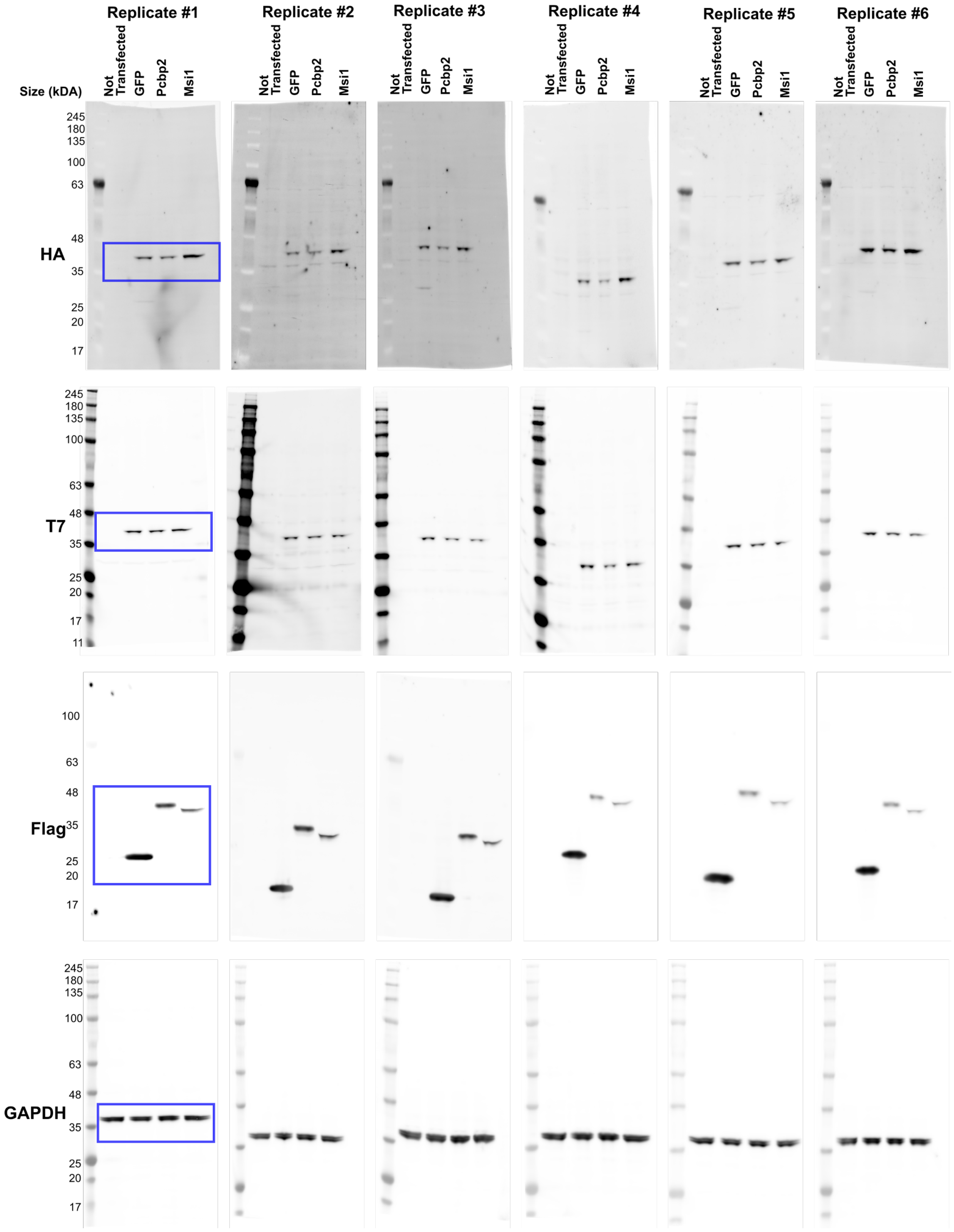

Supplementary Figure 13. Full size western blot images for the data presented on Figure 8C.

Labels on the left indicate the protein being probed. Blue boxes show the parts of the blots shown in Figure 8. The transfected samples include the construct indicated on the top along with expression constructs for HA-Gnat1 with wild type 3'-UTR and T7-Gnat1 with mutant 3'-UTR. Size markers indicate the molecular weight in kDA.
