# Supplementary material for: The Musashi proteins direct post-transcriptional control of protein expression and alternate exon splicing in vertebrate photoreceptors
Source: Commun Biol. 2022 Sep 24;5:1011. doi: 10.1038/s42003-022-03990-w (PMC9509328; doi:10.1038/s42003-022-03990-w)
Supplement: Supplementary file 3 — Description of Additional Supplementary Files [file 42003_2022_3990_MOESM3_ESM.pdf]

Description of Additional Supplementary Files

File name: Supplementary Data 1
Description: Differential RNA expression between retina with Msi1/Msi2 double knockout in photoreceptor cells and floxed controls. Column “Msi1 3'-UTR eCLIP” indicates that eCLIP peak is detected in the 3’-UTR

File name: Supplementary Data 2
Description: rMATS turbo output for cassette exons.

File name: Supplementary Data 3
Description: Non redundant set of cassette exons downregulated in the Msi1/Msi2 double knockout compared to floxed controls.

File name: Supplementary Data 4
Description: Non redundant set of cassette exons upregulated in the Msi1/Msi2 double knockout compared to floxed controls.

File name: Supplementary Data 5
Description: Differential protein expression between Msi1/Msi2 double knockout retina and floxed controls.

File name: Supplementary Data 6
Description: Gene Ontology and KEGG pathway enrichment analysis of proteins differentially expressed in photoreceptor double knockouts of Msi1 and Msi2 compared to floxed controls.

File name: Supplementary Data 7
Description: Gene Ontology and KEGG pathway enrichment analysis of proteins differentially expressed in photoreceptor double knockouts of Msi1 and Msi2 compared to floxed controls.

File name: Supplementary Data 8
Description: Proteomics and RNA-Seq data related to Figure 8. Column “Msi1 3'-UTR eCLIP” indicates that eCLIP peak is detected in the 3’-UTR.

File name: Supplementary Data 9
Description: Primers and probes used in this work.

File name: Supplementary Data 10
Description: Guide RNA sequences for generating photoreceptor specific exon knockouts

File name: Supplementary Data 11
Description: Antibodies used in this work.

File name: Supplementary Data 12
Description: Alternative exons used in metaexon analysis that are downregulated in the Msi1/Msi2 double knockout (upregulated by Musashi)

File name: Supplementary Data 13
Description: Alternative exons used in metaexon analysis that are upregulated in the Msi1/Msi2 double knockout (downregulated by Musashi)

File name: Supplementary Data 14
Description: Alternative exons used in metaexon analysis that remain unchanged in the Msi1/Msi2 double knockout

File name: Supplementary Data 15
Description: Data related to Figures 2, 4 and 8, and Supplementary Figures 2, 4 and 6

File name: Supplementary Data 16
Description: ERG data used in preparing Figure 6B. Scotopic (rod) and photopic (cone) A-wave recordings.

File name: Supplementary Data 17
Description: Peptide log2 signal intensities.

File name: Supplementary Data 18
Description: Normalized protein expression. Includes data used in the preparation of Figure 7.

File name: Supplementary Data 19
Description: BED files with MSI1 eCLIP cross-link sites and regions enriched over input.
