# Supplementary material for: The Musashi proteins direct post-transcriptional control of protein expression and alternate exon splicing in vertebrate photoreceptors
Source: Commun Biol. 2022 Sep 24;5:1011. doi: 10.1038/s42003-022-03990-w (PMC9509328; doi:10.1038/s42003-022-03990-w)
Supplement: Supplementary file 16 — Reporting Summary [file 42003_2022_3990_MOESM16_ESM.pdf]

nature portfolio

Corresponding author(s): Peter Stoilov

Last updated by author(s): Sep 1, 2022

Reporting Summary

Nature Portfolio wishes to improve the reproducibility of the work that we publish. This form provides structure for consistency and transparency in reporting. For further information on Nature Portfolio policies, see our Editorial Policies and the Editorial Policy Checklist.

Statistics

For all statistical analyses, confirm that the following items are present in the figure legend, table legend, main text, or Methods section.

n/a	Confirmed	
☐	☒	The exact sample size (*n*) for each experimental group/condition, given as a discrete number and unit of measurement
☐	☒	A statement on whether measurements were taken from distinct samples or whether the same sample was measured repeatedly
☐	☒	The statistical test(s) used AND whether they are one- or two-sided<br>*Only common tests should be described solely by name; describe more complex techniques in the Methods section.*
☐	☒	A description of all covariates tested
☐	☒	A description of any assumptions or corrections, such as tests of normality and adjustment for multiple comparisons
☐	☒	A full description of the statistical parameters including central tendency (e.g. means) or other basic estimates (e.g. regression coefficient) AND variation (e.g. standard deviation) or associated estimates of uncertainty (e.g. confidence intervals)
☐	☒	For null hypothesis testing, the test statistic (e.g. *F*, *t*, *r*) with confidence intervals, effect sizes, degrees of freedom and *P* value noted<br>*Give P values as exact values whenever suitable.*
☒	☐	For Bayesian analysis, information on the choice of priors and Markov chain Monte Carlo settings
☒	☐	For hierarchical and complex designs, identification of the appropriate level for tests and full reporting of outcomes
☒	☐	Estimates of effect sizes (e.g. Cohen's *d*, Pearson's *r*), indicating how they were calculated

Our web collection on statistics for biologists contains articles on many of the points above.

Software and code

Policy information about availability of computer code

Data collection	Data collection instruments, software and procedures are described in the Materials and Methods section
Data analysis	Software and procedures are described in the Materials and Methods, and Code Availability sections

For manuscripts utilizing custom algorithms or software that are central to the research but not yet described in published literature, software must be made available to editors and reviewers. We strongly encourage code deposition in a community repository (e.g. GitHub). See the Nature Portfolio guidelines for submitting code & software for further information.

Data

Policy information about availability of data

All manuscripts must include a data availability statement. This statement should provide the following information, where applicable:

- Accession codes, unique identifiers, or web links for publicly available datasets
- A description of any restrictions on data availability
- For clinical datasets or third party data, please ensure that the statement adheres to our policy

The eCLIP-Seq and RNA-Seq data is available at the NCBI Sequence Read Archive under project accessions PRJNA795195 and PRJNA795137. The mass spectrometry proteomics are deposited to the ProteomeXchange Consortium via the PRIDE partner repository with the dataset identifier PXD030748 and 10.6019/PXD030748

Human research participants

Policy information about studies involving human research participants and Sex and Gender in Research.

Reporting on sex and gender	NA
Population characteristics	NA
Recruitment	NA
Ethics oversight	NA

Note that full information on the approval of the study protocol must also be provided in the manuscript.

Field-specific reporting

Please select the one below that is the best fit for your research. If you are not sure, read the appropriate sections before making your selection.

☒ Life sciences ☐ Behavioural & social sciences ☐ Ecological, evolutionary & environmental sciences

For a reference copy of the document with all sections, see nature.com/documents/nr-reporting-summary-flat.pdf

Life sciences study design

All studies must disclose on these points even when the disclosure is negative.

Sample size	Sample sizes were not predetermiend
Data exclusions	No data was excluded
Replication	All experiments were reproduced as stated in the Materials and Mathods section
Randomization	Animal allocation was random using both sexes in comparable numbers. Animals in different groups were either litter-mates or were age matched
Blinding	Blinding was not possible because investigators were engaged in all aspects of the experiments

Reporting for specific materials, systems and methods

We require information from authors about some types of materials, experimental systems and methods used in many studies. Here, indicate whether each material, system or method listed is relevant to your study. If you are not sure if a list item applies to your research, read the appropriate section before selecting a response.

Materials & experimental systems

n/a	Involved in the study	
☐	☒	Antibodies
☐	☒	Eukaryotic cell lines
☒	☐	Palaeontology and archaeology
☐	☒	Animals and other organisms
☒	☐	Clinical data
☒	☐	Dual use research of concern

Methods

n/a	Involved in the study	
☒	☐	ChIP-seq
☒	☐	Flow cytometry
☒	☐	MRI-based neuroimaging

Antibodies

Antibodies used	Antibodies used are listed on supplementary table 11
Validation	Primary antibodies are validated by the manufacturers to be specific to the stated protein and species. In addition the Msi1, Msi2 and Prom1 antibodies were validated by us using animal knockout models.

Eukaryotic cell lines

Policy information about cell lines and Sex and Gender in Research

Cell line source(s)	Lab stock of NIH 3T3 cells
Authentication	not authenticated genetically, cell morphology matches ATCC data
Mycoplasma contamination	not tested
Commonly misidentified lines (See ICLAC register)	none

Animals and other research organisms

Policy information about studies involving animals; ARRIVE guidelines recommended for reporting animal research, and Sex and Gender in Research

Laboratory animals	Mus musculus. The strains are described in the materials and methods section
Wild animals	The study did not involve wild animals
Reporting on sex	Both sexes were used in comparable numbers.
Field-collected samples	The study did not involve field collected samples
Ethics oversight	Ethics oversight was provided by WVU IACUC. The studies were carried out under protocol numbers 2104041420 and 2104041420.1

Note that full information on the approval of the study protocol must also be provided in the manuscript.
